# Apigenin Attenuates Atherogenesis through Inducing Macrophage Apoptosis via Inhibition of AKT Ser473 Phosphorylation and Downregulation of Plasminogen Activator Inhibitor-2

**DOI:** 10.1155/2015/379538

**Published:** 2015-04-15

**Authors:** Ping Zeng, Bin Liu, Qun Wang, Qin Fan, Jian-Xin Diao, Jing Tang, Xiu-Qiong Fu, Xue-Gang Sun

**Affiliations:** ^1^Guangdong General Hospital, Guangdong Academy of Medical Sciences, Guangzhou 510080, China; ^2^Guangzhou Institute of Cardiovascular Disease, The Second Affiliated Hospital of Guangzhou Medical University, Guangzhou 510260, China; ^3^The Key Laboratory of Molecular Biology, State Administration of Traditional Chinese Medicine, School of Traditional Chinese Medicine, Southern Medical University, Guangzhou, Guangdong 510515, China; ^4^Nanfang Hospital, Southern Medical University, Guangzhou 510515, China; ^5^Center for Cancer and Inflammation Research, School of Chinese Medicine, Hong Kong Baptist University, Kowloon Tong, Hong Kong

## Abstract

Macrophage survival is believed to be a contributing factor in the development of early atherosclerotic lesions. Dysregulated apoptosis of macrophages is involved in the inflammatory process of atherogenesis. Apigenin is a flavonoid that possesses various clinically relevant properties such as anti-inflammatory, antiplatelet, and antitumor activities. Here we showed that apigenin attenuated atherogenesis in *apoE* ^−/−^ mice in an *in vivo* test. *In vitro* experiments suggested that apigenin induced apoptosis of oxidized low density lipoprotein- (OxLDL-) loaded murine peritoneal macrophages (MPMs). Proteomic analysis showed that apigenin reduced the expression of plasminogen activator inhibitor 2 (PAI-2). PAI-2 has antiapoptotic effects in OxLDL-loaded MPMs. Enhancing PAI-2 expression significantly reduced the proapoptosis effects of apigenin. Molecular docking assay with AutoDock software predicted that residue Ser473 of Akt1 is a potential binding site for apigenin. Lentiviral-mediated overexpression of Akt1 wild type weakened the proapoptosis effect of apigenin in OxLDL-loaded MPMs. Collectively, apigenin executes its anti-atherogenic effects through inducing OxLDL-loaded MPMs apoptosis. The proapoptotic effects of apigenin were at least partly attributed to downregulation of PAI-2 through suppressing phosphorylation of AKT at Ser473.

## 1. Introduction

Cardiovascular disease accounts for 32.3% (787,931) of all 2,437,163 deaths in 2009 in the United States. Atherosclerosis is the underlying cause of the majority of clinical cardiovascular events [1]. It is a systemic cardiovascular disease with complicated pathogenesis involving fat deposit, oxidative stress, endothelial dysfunction, chronic inflammation, and scar tissue buildup within the walls of arteries [2].

Atherosclerosis is an inflammatory disease [3]. Fatty streak, the earliest type of lesion which is common in infants and young children, is a pure inflammatory lesion consisting only of monocyte-derived macrophages and T lymphocytes. Macrophages are found in all stages of atherosclerosis and play pivotal role in the pathogenesis of atherosclerosis. Though the role of macrophages in the progression of atherosclerosis is still controversial, it has been reported that deficiency of p53 and Bax suppresses the apoptosis of macrophages and hence accelerates atherosclerosis progression [4, 5]. Moreover, increased apoptosis of macrophages reduces the size of early atherogenic lesions [6, 7]. Based on these observations, increased apoptosis of macrophage could reduce lesion size and subsequently attenuate the plaque progression.

Apigenin, a natural product that belongs to flavonoids, has been reported to induce apoptosis in human monocytic leukemia THP-1 [8] and human leukemia cell U937 [9]. Apigenin has been demonstrated to help in improving cardiovascular conditions, stimulating immune system, inhibiting platelet aggregation, and providing some protection against cancer [10, 11]. Our previous works showed that apigenin inhibited invasion and migration of colorectal cancer through inhibiting phosphorylation of AKT [12]. We thus want to know if apigenin has preventive effects on atherosclerosis through regulating macrophage mediated chronic inflammation, which is beyond the widely accepted “cholesterol hypothesis” [13].

Apigenin pretreatment inhibits oxidation of low density lipoprotein (LDL) [14] and blunts reactive oxygen species-triggered signaling pathway [15]. However, the effects of apigenin on atherosclerosis and the involved molecular mechanism have not been well studied yet. In our work, apolipoprotein E null (*apoE* ^−/−^) were was used to observe the effects of apigenin on atherogenesis. Oxidized LDL (OxLDL) treated murine peritoneal macrophages (MPMs) were used for analysis of the molecular mechanisms of apigenin. Our* in vivo* and* in vitro* studies provided direct evidences for the prevention of atherosclerosis with apigenin in a way of nutrition intervention.

## 2. Materials and Methods

### 2.1. Materials

Apigenin, dimethyl sulfoxide (DMSO), and thiazolyl blue tetrazolium bromide (MTT) were purchased from Sigma-Aldrich (St. Louis, MO, USA). RPMI 1640, FBS, and antibiotics were purchased from Invitrogen (Gibco, Grand Island, NY, USA). Oxygenized low density lipoprotein (OxLDL) was purchased from Yiyuan-Biotech (Guangzhou, China). Apoptosis Detection Kit was purchased from BD Biosciences (USA). AKT inhibitor MK2206 was bought from ApexBio (USA). Antibodies were bought from CST (Cell Signaling Technology, MA, USA) and Abcam (Cambridge, United Kingdom).

### 2.2. Animal Experiment

All procedures performed in studies involving animals were in accordance with the ethical standards of Animal Care Ethics Committee of Southern Medical University. The* apoE* ^−/−^ mice (Laboratorial Animal Center of Beijing University, Beijing, China) and C57BL/6 mice (Laboratory Animal Center of Southern Medical University, Guangzhou, China) are maintained under controlled conditions (22°C, 12-h/12-h dark/light cycle) in a conventional animal colony. Apigenin (10 mg) was suspended in 1 mL vehicle material (0.5% methyl cellulose and 0.025% Tween 20) by sonication for 30 s at 4°C [16]. 6-week-old mice were given apigenin (100 mg/kg/d) or simvastatin (1.53 mg/kg/d) and western diet (containing 20% fat and 0.15% cholesterol) feeding for consecutive 8 weeks. Treatment with simvastatin was served as a positive control. Mice intragastrically administered with vehicle material and feeding with western diet be served as the model, while mice feeding with general diet were served as the blank control. Mice were sacrificed at the end of the 8 weeks and aortas were separated.

### 2.3. Sudan III Stain and Immunohistochemistry

The degree of atherosclerosis development was assessed by Sudan III stain. The frozen sections of aortas were immersed in a solution of Sudan III (4% w/v in 70% alcohol) for 60 min. Distilled water was used to wash away excess Sudan III. Normally, adipose tissues would be seen to be stained orange red under the microscope.

Macrophage content was evaluated by monoclonal to monocyte + macrophage (MOMA-2) immunohistochemistry as previously described [17]. The image of each case was captured using a fluorescence microscope (Nikon Eclipse-Ti). Image analysis was performed using Image-Pro Plus 6.0 (IPP6) software.

### 2.4. MPMs Culture and Cell Viability

The C57BL/6 mice were euthanized and the abdominal cavity was lavaged with 8 mL of ice-cold RPMI 1640 to collect resident peritoneal macrophages. The macrophages were cultured in RPMI 1640 with 5% FBS and 1% penicillin and streptomycin. After 4 hours, nonadherent cells were washed away with PBS. Adherent cells were then treated with OxLDL in present/absent of apigenin. Cell viability was determined by the MTT assay.

### 2.5. Annexin V/PI Assay

To determine the extent of apoptosis and necrosis of macrophage-derived foam cells, cells were double stained with Annexin V/PI staining (FITC Annexin V Apoptosis Detection Kit I, BD Pharmingen, USA) assayed with FACS Calibur flow cytometer (BD Biosciences, USA).

### 2.6. Molecular Docking

Molecular docking can fit molecules together in a favorable configuration to form a complex system. The structural information from the theoretically modeled complex may help us to clarify the binding mechanism between Akt (PDB: 4GV1, this 3D crystal structure of Akt is replaced serine with aspartic acid in position 473 for simulation of Ser473 phosphorylation) and apigenin (CID: 5280443). We performed automated docking study by using AutoDock v4.2. The Lamarckian Genetic Algorithm (LGA) was applied to docking simulation and the distance-dependent dielectric constant function was used for the calculation of energetic maps. Interaction energies were calculated for various docked positions and ranked in accordance with the interaction energies between the ligand and the protein.

### 2.7. 2-DE, Gel Staining, Tryptic Digestion of 2-DE Gel Spots, and Mass Spectrometry (MS) Identification

Cells were washed in PBS and resuspended in lysis buffer (50 mM Tris (pH 7.5), 5 mM EDTA, 10 mM EGTA, 50 mM NaF, 20 mM *β*-glycerophosphate, 250 mM NaCl, 2% NP-40, and protease inhibitors). Protein extraction, 2-DE, gel staining, tryptic digestion of 2-DE gel spots, and mass spectrometry (MS) identification were performed as previously described according to standard protocols [18].

### 2.8. Western Blotting

The phosphorylated protein extraction was performed using the FOCUS PhosphoRich Kit (G-Bioscience, USA) according to the instructions. Western blotting was performed as previously described. Briefly, equal amounts of protein lysate were separated on 12% SDS gel and then electrotransferred to the PVDF membrane. The membrane was incubated with the different primary antibodies: anti-AKT (Cell Signaling Technology, CST, 1 : 1000), anti-phospho-Akt (Ser473, CST, 1 : 1000), and anti-PAI-2 (Abcam, 1 : 1000 dilution) over night at 4°C. Horseradish peroxidase signals were detected by enhanced chemiluminescence substrate (ECL Plus Western Blotting Detection System, GE Healthcare, Uppsala, Sweden) and captured with a CCD system (image station 2000MM, Kodak, Rochester, NY, USA). The quantitative analysis of these images was performed using Molecular Imaging Software Version 4.0, provided by Kodak 2000MM System [19]. The optical density was normalized against that of *β*-actin.

### 2.9. siRNA and Transient Transfection

For knocking down PAI-2, three PAI-2 siRNA oligos (PAI2-216, PAI2-763, and PAI2-821) and control siRNA oligo (NC) were purchased from Gene Pharma (Shanghai, China). The cells at 60–70% confluence were transfected with plasmids or siRNA oligos using Lipofectamine 2000 and Plus reagents from Invitrogen as described previously [19].

### 2.10. Lentivirus Production and Cell Transduction

The human AKT1 (NM_001014431.1) and human PAI-2 (NM_001143818.1) were constructed by initially using a PCR strategy for cloning into the Hpa I/XbaI and Hpa I/Xma I site of the pHIV-H2BmRFP lentiviral vector, respectively. AKT S473A mutant was generated by PCR with MutanBEST Kit (Takara, Dalian, China) as described [20]. All clones were sequenced to verify the authenticity of the gene or the mutant. The primers sequences are shown in Table 1. To produce recombinant lentivirus, lentiviral vectors were cotransfected with psPAX2 and pMD2Ginto HEK 293T cells with Lipofectamine 2000. Lentivirus was harvested at about 48 h after transfection. The final titer of recombinant virus was adjusted to 1 × 10^7^ TU/mL.

### 2.11. Statistical Analysis

Data are expressed as the mean ± S.E.M. for each experimental group. All the data were analyzed using the SPSS statistical package (version 13.0, SPSS Inc., Chicago, IL, USA). Univariate analysis was performed using one-way ANOVA, factorial analysis. The differences between two groups were analyzed, based on the test of least significance difference (LSD). If the variances were not homogenous, mean values were compared using Welch's test. The differences between two groups were analyzed by Dunnett's T3. *P* < 0.05 was considered statistically significant.

## 3. Results

### 3.1. The Impact of Apigenin on Atherogenesis in Mice

**Figure 1:**
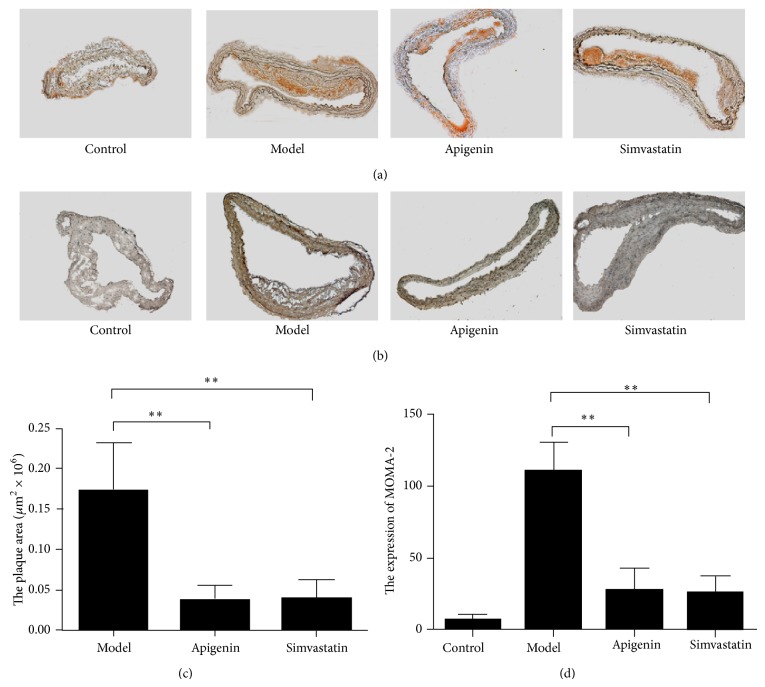
Antiatherogenesis activity of apigenin in apo*E* ^−/−^ *mice*. (a) Representative images of the aortas stained with Sudan III. (b) Representative images of MOMA-2 stained sections from the proximal aortas. (c) Quantitative analyses of the plaque areas in (a). (d) Quantification of MOMA-2 expression level in (b). ^∗∗^ *P* < 0.01 compared with the model group.

To determine whether apigenin can prevent the development of vascular lesions, apigenin and a western diet were started simultaneously in 6-week-old* apoE* ^−/−^ mice in an early intervention study. Staining of cross sections of the proximal aorta with Sudan III showed the smaller atherosclerotic lesion area in the proximal aorta in the apigenin-treated group as compared with the model group (42.8%, *P* < 0.01) (Figures 1(a) and 1(c)). As the constitution of fatty streak was almost exclusively macrophage-derived foam cells, macrophages determined by MOMA-2 were located predominantly on the luminal surface of the lesions. The immunohistochemistry results showed that the aorta infiltrating macrophages were significantly reduced in the apigenin-treated group (Figures 1(b) and 1(d)).

### 3.2. Apigenin Reversed the Prosurvival Effects of OxLDL and Induced Apoptosis of MPMs

**Figure 2:**
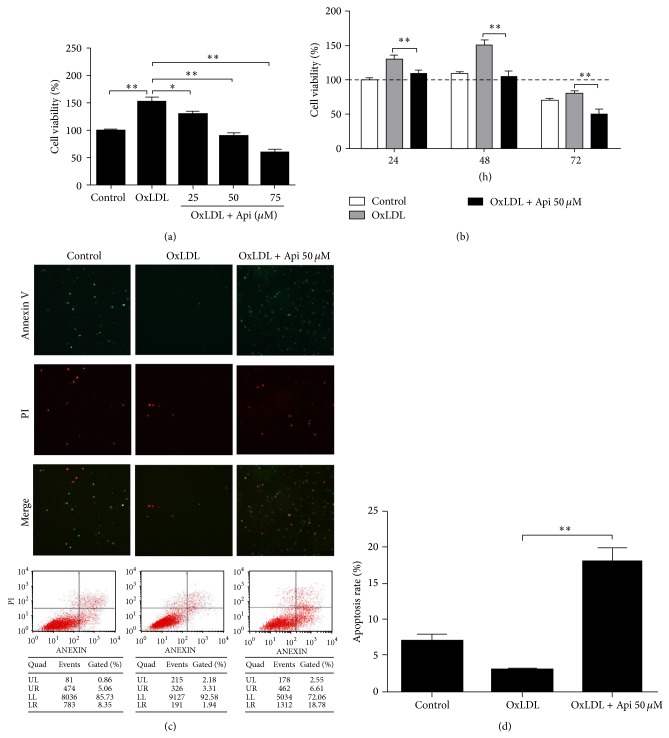
Protective effects of oxidized LDL and native LDL on macrophages. Murine peritoneal macrophages were plated at a density of 1 × 10^4^ cells/well in 96 well plate. Then cells were treated with the indicated concentration of apigenin for 48 h after 1 h preincubation with 50 *μ*g/mL OxLDL. Cell viability was measured by MTT assay. (a) Apigenin significantly reduced the viability of MPM in a dose dependent manner. ^∗^ *P* < 0.05, ^∗∗^ *P* < 0.01 compared with the control. (b) Apigenin significantly reduced the viability of MPM pretreated with OxLDL in a time dependent manner. ^∗^ *P* < 0.05, ^∗∗^ *P* < 0.01 compared with the control. ((c)-(d)) Apigenin significantly increased the apoptosis of MPM. Each value represents the mean ± S.E.M of quadruplicate determinations from three independent experiments.

Taking up of oxidized LDL by macrophages induces foam cell formation. The process is unregulated and ultimately generates a potent proinflammatory milieu [21]. OxLDL has been shown to promote macrophage proliferation and inhibit apoptosis at lower concentrations [22]. In our study, no obvious proliferation was observed in phorbol 12-myristate-13-acetateprimed THP-1 cells pretreated with OxLDL (see Figure S1 in Supplementary Material available online at http://dx.doi.org/10.1155/2015/379538). However, the MTT results showed that OxLDL significantly increased survival rate of MPMs (Figure 1(a)). The increasing cell viability induced by OxLDL was not due to the proliferation of MPMs (Figures S2 and S3). Apigenin significantly decreased cell viability in a dose- and time-dependent manner in MPMs pretreated with OxLDL (*P* < 0.05) (Figures 2(a) and 2(b)). Apigenin 50 *μ*M treatment for 48 h was effective in reducing viability of OxLDL-loaded MPMs and thus was chosen in the following study. We further tested the proapoptosis effect of apigenin using flow cytometry. Our results suggested that apigenin 50 *μ*M induced OxLDL-loaded MPMs apoptosis (Figures 2(c) and 2(d)). Comparing to the control group, apoptosis ratio increased to 55.49% when MPMs were treated with 50 *μ*M apigenin for 48 h.

### 3.3. Effects of Apigenin on Proteomic Changes

**Figure 3:**
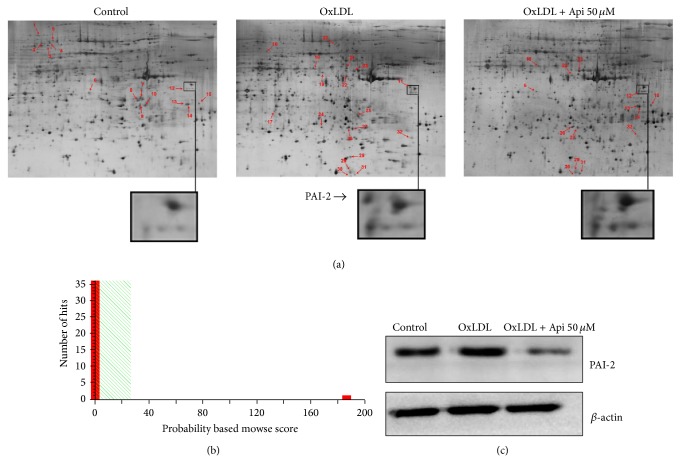
Proteomic differences between murine peritoneal macrophages incubated in the absence or presence of apigenin after stimulated by* OxLDL*. Cells were treated with/without 50 *μ*M apigenin for 48 h after 1 h pretreatment with 50 *μ*g/mL OxLDL. (a) Representative two-dimensional gel electrophoresis (2DE) gels showing the locations of spots with different intensities in control, OxLDL, and apigenin + OxLDL groups are shown. Protein extraction, 2-DE, and gel staining were performed as previously described. (b) Spot 11 was identified as PAI-2 according to the MOWSE score. (c) Representative results of Western blotting that PAI-2 were significantly increased by OxLDL, but decreased by apigenin. *β*-actin was used as a loading control. Results shown are the representative of three independent experiments.

To further explore the involved molecules in apigenin-induced apoptosis in OxLDL-loaded MPMs, two-dimensional electrophoresis (2-DE) and mass spectrometry were performed as described previously [23]. Twenty-eight proteins were identified successfully including fourteen downregulated spots and fourteen upregulated spots caused by OxLDL (Figure 3(a)). Among them, four proteins including V-type proton ATPase subunit d 1, heterogeneous nuclear ribonucleoprotein K, and Annexin A5 were downregulated by OxLDL, but upregulated by apigenin; eight proteins including plasminogen activator inhibitor 2 (PAI-2), tyrosine-protein phosphatase nonreceptor type 6, arginase-1, cytochrome b-c1 complex subunit 1, and alpha-enolase were upregulated by OxLDL, but downregulated by apigenin.  Figure 3(b) showed that spot 11 was identified as PAI-2 according to the MOWSE score. The 2D results indicated that PAI-2 could be upregulated by OxLDL, and downregulated apigenin in MPMs. Western blot analysis further confirmed the 2-DE expression pattern of PAI-2 (Figure 3(c)).

### 3.4. Apigenin Induced Apoptosis of MPM through Downregulating PAI-2

**Figure 4:**
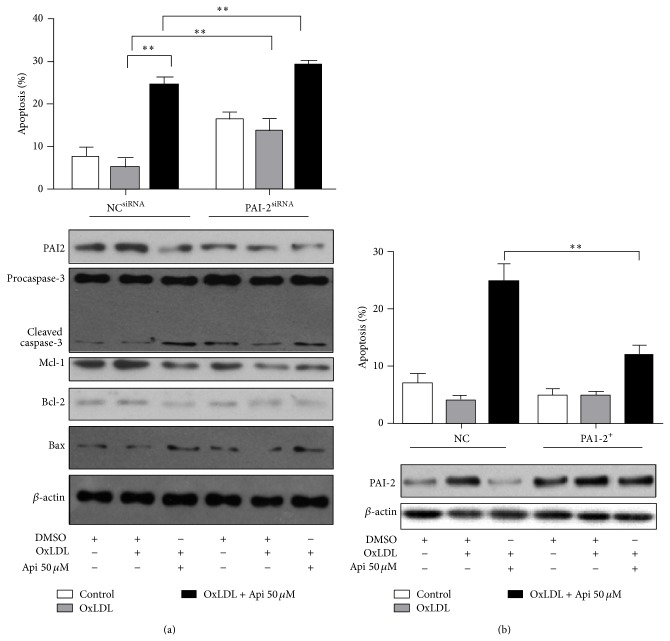
PAI-2 inhibited apoptosis* of MPM*. (a) PAI-2 siRNA increased apoptosis of MPMs. After 24 hours of transfection with siRNA against PAI-2, cells were treated with apigenin (50 *μ*M) or DMSO (0.1%) for 48 h after 1 h pretreatment with OxLDL (50 *μ*g/mL). Protein expressions of PAI-2, caspase-3, Mcl-1, Bcl-2, and Bax expression were examined by Western blotting. *β*-actin was included as a loading control. (b) PAI-2 overexpression lowered the apoptosis rate induced by apigenin. The protein expression level of PAI-2 was examined by Western blotting. Results of Western blotting shown in (a) and (b) are the representative of three independent experiments. MPM apoptosis was determined by flow cytometric analysis of cells double stained with Annexin V/PI. The percentage of apoptotic cells was presented as the mean ± S.E.M of three independent experiments. ^∗∗^ *P* < 0.01.

Three PAI-2 siRNAs were prescreened (Figure S4) and siRNA PAI2-216 was chosen for the highest silencing efficiency. It increased the apoptosis rate (lane 5) as compared to the control siRNA (lane 2) in OxLDL-loaded MPMs. It also exacerbated the apoptosis rate induced by apigenin (lane 6) as compared to the control siRNA (lane 3). It increased the expression of cleaved caspase-3 corresponding to the apoptosis rate (Figure 4(a)). Apigenin also suppressed the antiapoptotic proteins, Mcl-1 and Bcl-2, and increased Bax in MPMs (lane3). However, the regulation of Mcl-1, Bcl-2 and Bax by apigenin might be not mediated by PAI-2 (lanes 4, 5, and 6).

To further confirm the role of PAI-2 in apigenin induced apoptosis, OxLDL loaded-MPMs were infected with lentivirus containing PAI-2. PAI-2 overexpression lowered the apoptosis rate induced by apigenin (Figure 4(b)). Taken together, PAI-2 is at least partly responsible for apigenin induced apoptosis in OxLDL-loaded MPMs.

### 3.5. Akt Ser473 Is a Potential Target for Regulating PAI-2

**Figure 5:**
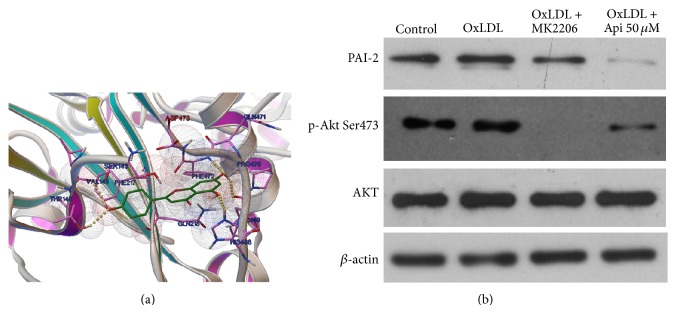
Molecular docking analysis of the interaction between apigenin and* Akt*. (a) Molecular docking analysis of the interaction between apigenin and Akt. (b) Apigenin lowered the expression of PAI-2 and phosphorylation of Akt Ser473. Cells were treated with apigenin (50 *μ*M) or DMSO (0.1%) for 48 h after 1 h pretreatment with OxLDL (50 *μ*g/mL). The Akt inhibitor (MK2206, 4 *μ*M) was used as a positive control. Proteins from cultured cells were extracted for Western blotting. Results shown are the representative of three independent experiments. *β*-actin was used as a loading control.

To search the potential target for apigenin, the interaction between apigenin and Akt was analyzed by homology modeling with AutoDock software [24]. The docking result showed that apigenin can be docking with Akt Ser473, and the best fit binding energy and estimated inhibition constant (Ki) for the apigenin-Akt Ser473 interaction were −6.64 kcal/mol and 13.63 *μ*M, respectively (Figure 5(a)). The optimal binding conformation of the Akt-apigenin complex was accordingly selected. The effect of apigenin on phosphorylation Ser473 in OxLDL-loaded MPMs was verified subsequently. Western blotting results showed that OxLDL increased the phosphorylation of Akt Ser473, and apigenin decreased the OxLDL-upregulated phosphorylation level (Figure 5(b)). To explore the role of Akt in PAI-2 regulation, we used an Akt inhibitor (MK2206) as a positive control. The reduction of PAI-2 expression (Figure 5(b)) by MK2206 suggested that PAI-2 might be partially regulated by Akt.

### 3.6. Apigenin Induced OxLDL-Loaded MPMs Apoptosis through Regulating Akt and PAI-2

**Figure 6:**
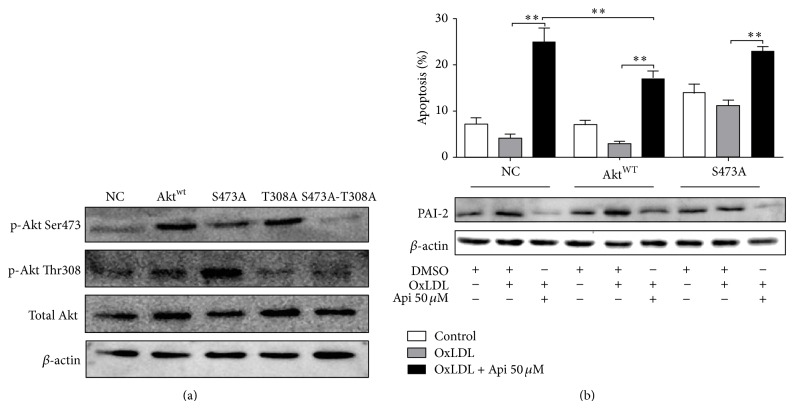
Overexpression of AKT lowered the apoptosis rate induced by apigenin. (a) After 72 h lentiviral transfection of empty vector, Akt wild type, AktS473A, Akt T308A, or AktS473A-T308A, proteins from MPM were extracted for Western blotting. (b) After lentiviral transfection with empty vector, Akt wild type, or AktS473A, cells were treated with apigenin (50 *μ*M) or DMSO (0.1%) for 48 h after 1 h pretreatment with OxLDL (50 *μ*g/mL) and subjected to flow cytometric analysis of cells double stained with Annexin V/PI. Proteins from MPM were extracted for Western blotting. Akt wild type increased the expression of PAI-2 and inhibited the apoptosis induced by apigenin (^∗^ *P* < 0.05, ^∗∗^ *P* < 0.01). Results shown are the representative of three independent experiments.

5 lentivirus containing empty vector, Akt wild type (WT), 473 serine to glycine (S473G), 308 threonine to glycine (T308G), and double mutant of 473 serine and 308 threonine (S473G-T308G) were constructed and prepared as described previously (Figure S5) [12, 25] (Figure 6(a)). To determine the role of Akt Ser473 phosphorylation in regulating apigenin induced apoptosis, the apoptosis of MPMs pretreated with lentivirus containing empty control vector (negative control, NC), AktWT, and S473A were analyzed. As show in Figure 6(b), treatment with AktWT lentivirus increased the phosphorylation of Akt Ser473 and the expression of total Akt. Treatment with AKT S473A increased the total Akt level, but did not affect the phosphorylation of Akt Ser473. AktWT inhibited apigenin induced apoptosis and S473A increased apoptosis rate. Correspondingly, AktWT upregulated the expression of PAI-2 and S473A lowered the PAI-2 expression level. These results indicated that apigenin induces apoptosis through Akt dependent and independent regulation of PAI-2 (Figure 6(b)).

## 4. Discussion

Fatty streaks initially consist of lipid-laden monocytes and macrophages (foam cells) together with T lymphocytes. The presence of macrophage foam cells in the arteries of healthy young adults identified this disease as an unusual form of chronic inflammation [26]. Counteracting inflammation in human macrophages is a potential therapeutic implication in atherosclerosis [27]. It has been reported that apigenin inhibits the uptake of OxLDL and OxLDL induced endothelial dysfunction [28] and attenuates the secretion of nitric oxide and tumor necrosis factor-*α* [29]. Consistent with the report that antioxidant polyphenols have antiatherosclerotic effects [30], our* in vivo* test demonstrated that apigenin, a polyphenolic compound, has antiatherosclerotic effects in the* apoE* ^−/−^ mice receiving western diet.

Macrophage survival and proliferation are believed to be a contributing factor in the development of early atherosclerotic lesions [31]. Macrophage apoptosis does not induce the development of atherosclerosis because these cells are cleared by efferocytosis in early lesions [7]. It has been reported that accelerated apoptosis of macrophage resulted in reduced lesion size and a subsequent attenuation of plaque progression [4, 6, 7, 32, 33]. OxLDL has been reported to increase the viability of macrophage by directly activating a PI 3-kinase/PKB-dependent pathway [34]. In our study, apigenin exhibited a proapoptotic effect on OxLDL-loaded MPMs in a dose dependent manner and increased the expression of pro-apoptotic Bax, cleaved caspase-3, while it decreased the antiapoptotic Mcl-1 and Bcl-2. These results suggested that the antiatherosclerosis effects of apigenin are associated with the upregulation of apoptosis in OxLDL-loaded MPMs.

PAI-2 is a member of the ovalbumin-serpin subfamily (SERPIN clade B) based on gene structure and amino acid sequence homology. PAI-2 exists as a predominantly intracellular nonglycosylated 47 kDa-protein as well as a secreted glycosylated 60 kDa protein [35]. The expression of PAI-2 was augmented by OxLDL in the macrophages and high protein synthesis of PAI-2 was found in atherosclerotic lesions [36–38]. In addition, previous study showed that upregulation of PAI-2 inhibited the TNF-induced apoptosis in HeLa cells and limited caspase-3 activation [39, 40]. In our study, protein expression of PAI-2 was upregulated by OxLDL in MPMs. It has been reported that overexpressed PAI-2 protects cells from apoptosis in various models [41, 42], including human macrophages exposed to pathogens [43, 44]. Our results by studying with siRNA and overexpression of PAI-2 further provide the evidence that PAI-2 has antiapoptotic effects in OxLDL-loaded MPMs. These results suggested that downregulation of PAI-2 was involved in apigenin induced apoptosis of MPMs.

Apigenin has been shown to downregulate the phosphorylation level of Akt (Ser473) in cell line U937 [45]. Another study also shows that apigenin attenuates tumor growth in U937 xenografts through inactivation of Akt [9]. But the direct interaction of structure between apigenin and Akt remains unknown. For such interaction studies, the most important requirement was the proper orientation and conformation of ligand which should fit to the binding site of protein and form protein-ligand complex. Therefore, the optimal interactions and the best AutoDock score generated by AutoDock v4.2 software were used as criteria to interpret the best conformation. Lentivirus mediated AktWT and S473A overexpression in OxLDL-loaded MPMs indicated that phosphorylation of Akt Ser473 plays a critical role in apigenin induced apoptosis of OxLDL-loaded MPMs.

Akt phosphorylation promotes cellular survival through enhancing the antiapoptosis genes [46]. OxLDL synchronously upregulated the expression of PAI-2 and phosphorylation of AKT Ser473. In our study, OxLDL could not increase PAI-2 expression in S473A mutant MPMs, while Akt inhibitors could decrease PAI-2 in Akt wile type MPMs. These results suggested that phosphorylation of Akt Ser473 plays an important role in OxLDL induced PAI-2 activation in MPMs. Furthermore, overexpression of AktWT and S473Ain MPMs weakened and strengthened the proapoptotic effects of apigenin in MPMs, respectively. Therefore, apigenin regulates the expression of PAI-2 through an Akt Ser473 dependent pathway that induced apoptosis in OxLDL-loaded MPMs.

In summary, we have demonstrated that apigenin prevented atherogenesis in* apoE* ^−/−^ mice through inducing OxLDL-loaded MPMs apoptosis. The proapoptotic effects of apigenin were at least partly attributed to downregulation of PAI-2 through surpressing phosphorylation of AKT at Ser473.

## Supplementary Material

OxLDL significantly increased survival rate of MPMs. However, no obvious increased cell viability was observed in phorbol 12-myristate-13-acetateprimed THP-1 cells by pretreated with OxLDL (Figure S1). In MPMs, the increasing cell viability induced by OxLDL was not due to the cell proliferation (Figure S2, S3). PAI-2 siRNAs (Figure S4) and lentivirus (Figure S5) containing empty vector, Akt wild type (WT), 473 serine to glycine (S473G), 308 threonine to glycine (T308G), and double mutant of 473 serine and 308 threonine (S473G-T308G) were constructed and used to study the role of PAI-2 and Akt S473 in the apigenin induced apoptosis of OxLDL loaded MPMs.

## Figures and Tables

**Table 1:** MALDI-TOF MS identification of protein molecules with altered expression in OxLDL treated MPM compared with OxLDL + apigenin treated MPM.

Spot number^a^	Accession number	Protein score (threshold)^b^	Molecular weight^c^	Calculated pI value^c^	Name of protein	Expression level^d^

1	Q8CAQ8 (IMMT_MOUSE)	38 (27)	84247	6.18	Mitochondrial inner membrane protein	↓
2	P12265 (BGLR_MOUSE)	55 (28)	74648	6.22	Beta-glucuronidase	↓
3	Q61107 (GBP4_MOUSE)	74 (25)	71270	6.23	Guanylate-binding protein 4	↓
4	P12265 (BGLR_MOUSE)	143 (28)	74648	6.22	Beta-glucuronidase	↓
5	Q91ZA3 (PCCA_MOUSE)	80 (27)	80498	6.83	Propionyl-CoA carboxylase alpha chain, mitochondrial	↓
6	P58252 (EF2_MOUSE)	264 (27)	96222	6.41	Elongation factor 2	↓
7	N/A^e^					↓
8	P60710 (ACTB_MOUSE)	79 (26)	42052	5.29	Actin, cytoplasmic 1	↓
9	P60710 (ACTB_MOUSE)	107 (26)	42052	5.29	Actin, cytoplasmic 1	↓
10	Q9DB05 (SNAA_MOUSE)	106 (26)	33624	5.30	Alpha-soluble NSF attachment protein	↓
11	P12388 (PAI2_MOUSE)	187 (26)	46376	5.04	Plasminogen activator inhibitor 2, macrophage	↑, *⇓*
12	P51863 (VA0D1_MOUSE)	198 (27)	40731	4.89	V-type proton ATPase subunit d 1	↓, *⇑*
13	P61979 (HNRPK_MOUSE)	47 (27)	51230	5.39	Heterogeneous nuclear ribonucleoprotein K	↓, *⇑*
14	P48036 (ANXA5_MOUSE)	192 (28)	35787	4.82	Annexin A5	↓, *⇑*
15	P17918 (PCNA_MOUSE)	133 (28)	29108	4.66	Proliferating cell nuclear antigen	↓, *⇑*
16	P29351 (PTN6_MOUSE)	134 (28)	67859	7.66	Tyrosine-protein phosphatase nonreceptor type 6	↑, *⇓*
17	Q61176 (ARGI1_MOUSE)	37 (27)	34957	6.52	Arginase-1	↑, *⇓*
18	P46471 (PRS7_MOUSE)	84 (27)	49016	5.72	26S protease regulatory subunit 7	↑
19	P07724 (ALBU_MOUSE)	102 (26)	70700	5.75	Serum albumin	↑, *⇓*
20	Q99K51 (PLST_MOUSE)	138 (27)	71210	5.42	Plastin-3	↑
21	Q8JZX4 (SPF45_MOUSE)	42 (26)	45504	5.65	Splicing factor 45	↑
22	Q60854 (SPB6_MOUSE)	138 (27)	42913	5.53	Serpin B6	*⇑*
23	Q9CZ13 (QCR1_MOUSE)	122 (27)	53420	5.81	Cytochrome b-c1 complex subunit 1, mitochondrial	↑, *⇓*
24	P17182 (ENOA_MOUSE)	189 (26)	47453	6.37	Alpha-enolase	↑, *⇓*
25	Q9D154 (ILEUA_MOUSE)	171 (27)	42719	5.85	Leukocyte elastase inhibitor A	↑, *⇓*
26	Q9CQ60 (6PGL_MOUSE)	226 (26)	27465	5.55	6-phosphogluconolactonase	↑
27	P60710 (ACTB_MOUSE)	304 (26)	42052	5.29	Actin, cytoplasmic 1	↑
28	P11352 (GPX1_MOUSE)	75 (26)	22544	6.74	Glutathione peroxidase 1	↑, *⇓*
29	P09528 (FRIH_MOUSE)	121 (28)	21224	5.53	Ferritin heavy chain	↑

Notes: ^a^defined according to spot positions in 2-DE gel indicated, as in Figure 3(a).

^b^Protein scores are derived from ions scores as a nonprobabilistic basis for ranking protein hits. Protein scores greater than threshold value are significant (*P* < 0.05).

^c^Molecular weight value calculated by amino acid count; pI value calculated from the database entry without any processing.

^d^↑↓ Expression of OxLDL group compared to that of vehicle group; *⇑⇓* expression of apigenin group compared to that of OxLDL group.

^e^No effective peaks of peptide mass fingerprinting were available in spot 7.
