# Supplementary material for: Apigenin Attenuates Atherogenesis through Inducing Macrophage Apoptosis via Inhibition of AKT Ser473 Phosphorylation and Downregulation of Plasminogen Activator Inhibitor-2
Source: Oxid Med Cell Longev. 2015 Apr 15;2015:379538. doi: 10.1155/2015/379538 (PMC4413885; doi:10.1155/2015/379538)
Supplement: Supplementary file 1 — OxLDL significantly increased survival rate of MPMs. However, no obvious increased cell viability was observed in phorbol 12-myristate-13-acetateprimed THP-1 cells by pretreated with OxLDL (Figure S1). In MPMs, the increasing cell viability induced by OxLDL was not due to the cell proliferation (Figure S2, S3). PAI-2 siRNAs (Figure S4) and lentivirus (Figure S5) containing empty vector, Akt wild type (WT), 473 serine to glycine (S473G), 308 threonine to glycine (T308G), and double mutant of 473 serine and 308 threonine (S473G-T308G) were constructed and used to study the role of PAI-2 and Akt S473 in the apigenin induced apoptosis of OxLDL loaded MPMs. [file 379538.f1.docx]

**Supplementary Materials**

**Figure S1**

**The effects of Apigenin on the viability and apoptosis of OxLDL primed THP-1 cells.**

THP-1 cells primed with phorbol 12-myristate-13-acetate (PMA) were differentiated into macrophages. The macrophages pretreated with the indicated concentrations of OxLDL were dealed with apigenin (50μM) or DMSO (0.1%) for 48h. (A) The viability of THP-1 measured by MTT assay; (B-C) The apoptosis of THP-1 measured by ﬂow cytometry with annexin V-FITC-PI staining.


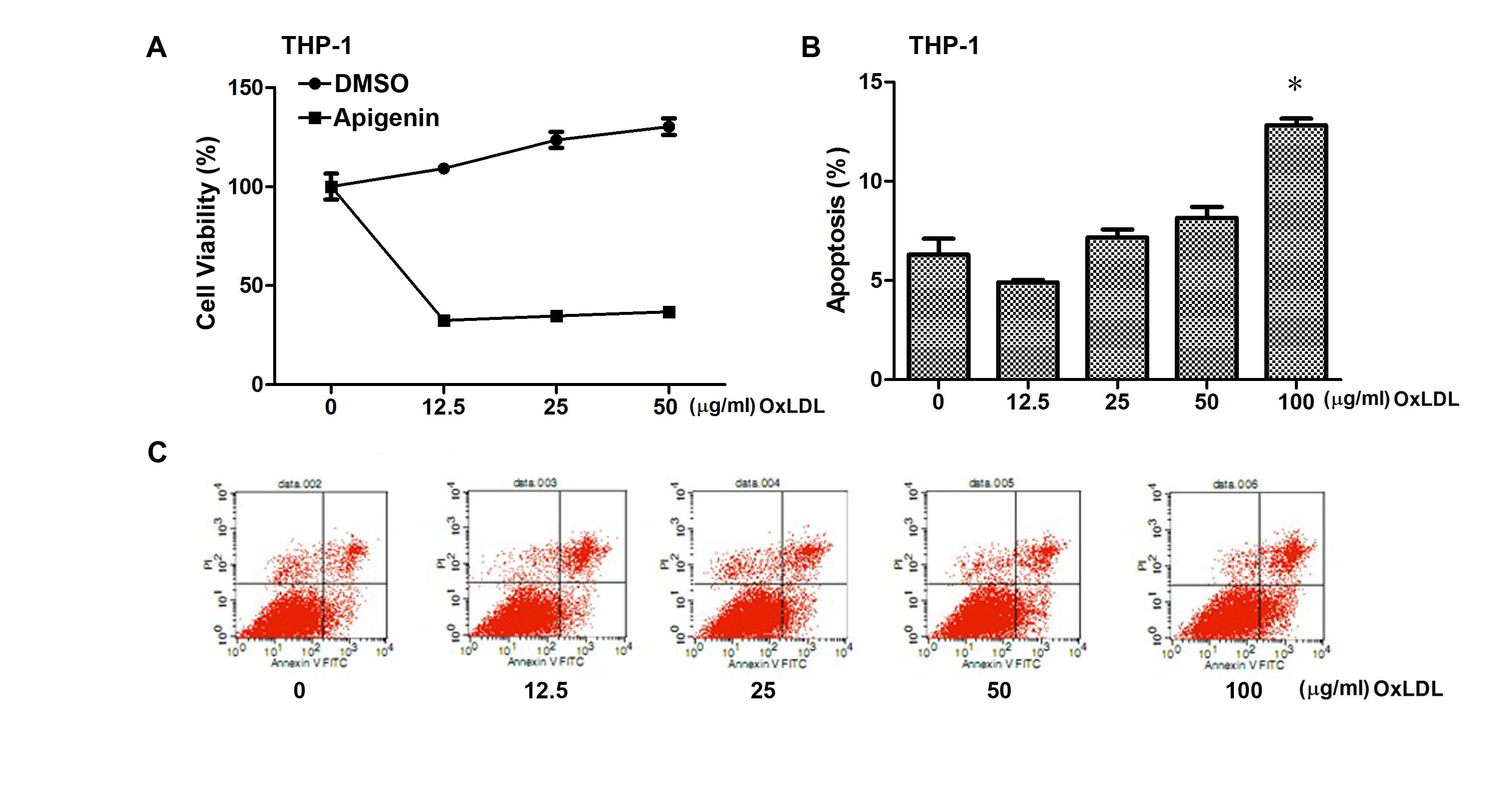


**Figure S2**

**Identification of primary murine peritoneal macrophages.**

# Murine peritoneal macrophages were identified using a phycoerythrin (PE)-conjugated speciﬁc anti-F4/80 antibody (BD Biosciences, USA) by ﬂow cytometry. The cells at upper area are defined as macrophages.


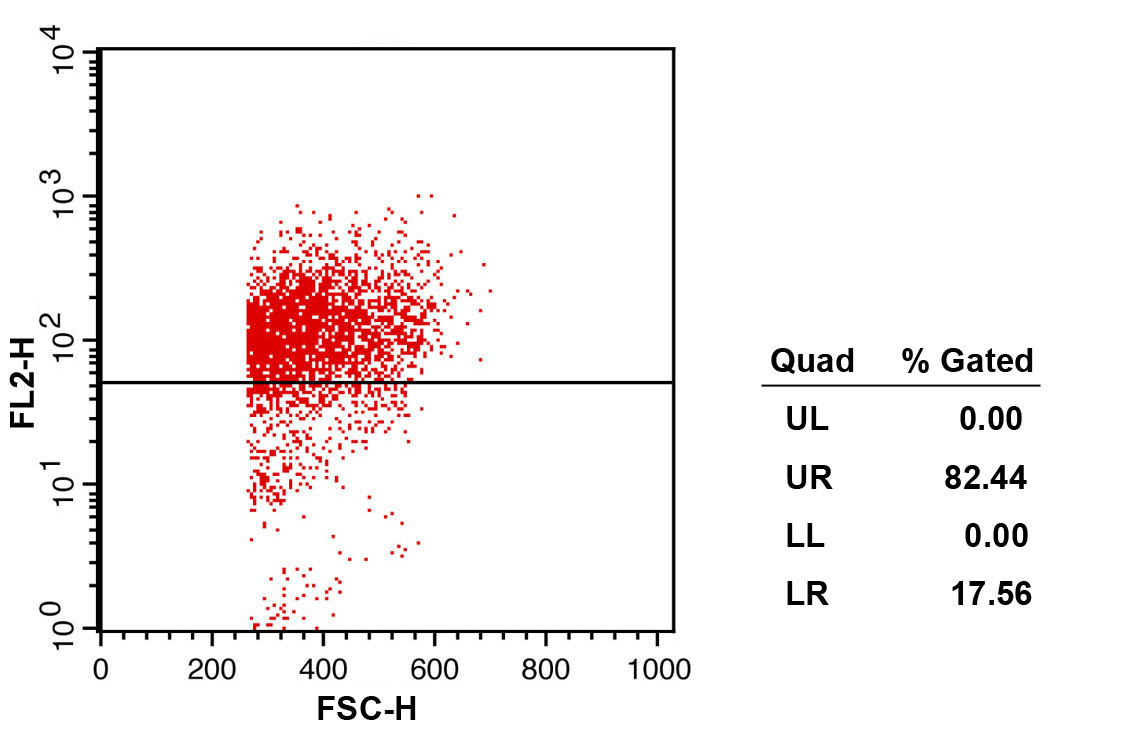


# Figure S3

**OxLDL did not increase murine peritoneal macrophages proliferation.**

Murine peritoneal macrophages were pretreated with OxLDL (50μg/ml) and then treated with apigeini (50μM) or DMSO (0.1%) for 48h. Cells were stained with EdU (marker of DNA synthesis) and heochst (stain for cell nucleus). Ls174T human colon cancer cells were used as a positive control.


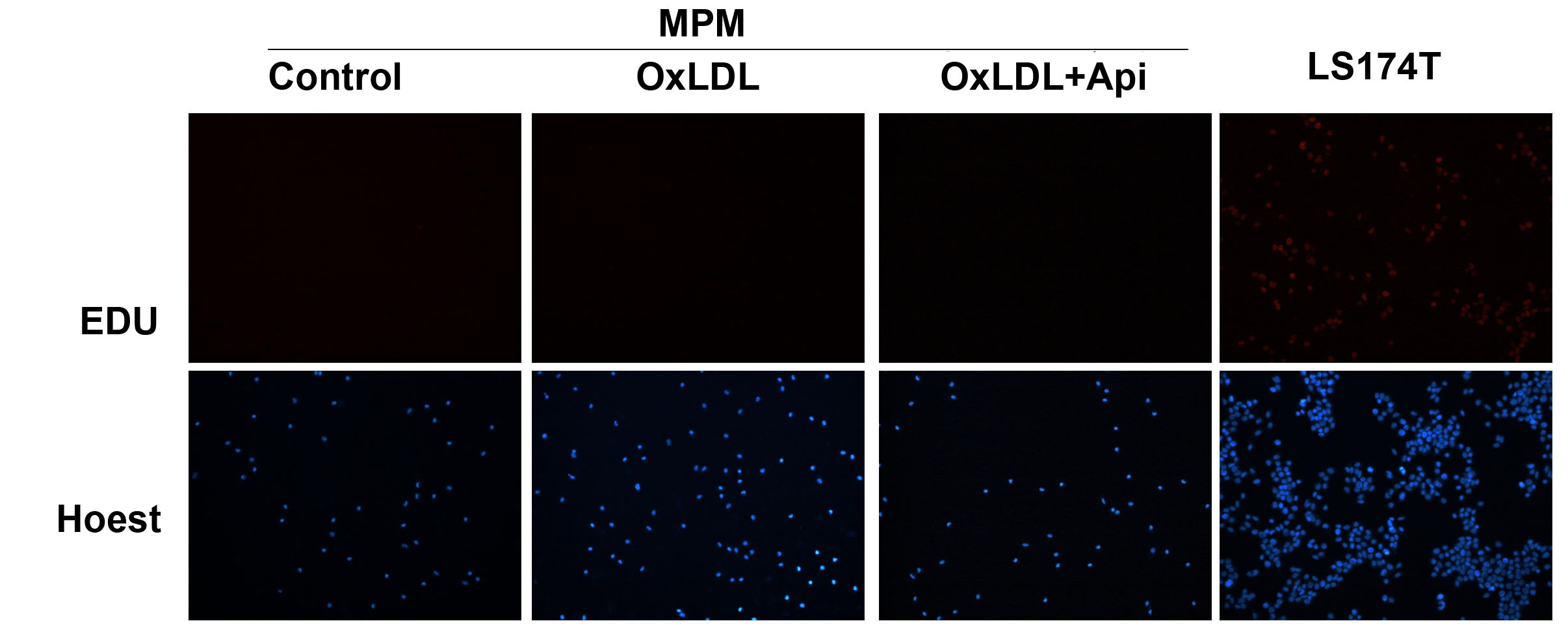


# Figure S4

# Screening efficient siRNA for PAI-2 on murine peritoneal macrophages .

# Murine peritoneal macrophages were transfected with siRNA oligonucleotides against PAI2-216, PAI2-763, and PAI2-821. 24 hours after transfection, the effects of siRNAs on PAI-2 expression were assessed by quantitative real-time PCR. Data (^∆∆^Ct ) are mean ± SD, n = 3, # *P*<0.05 *vs* control (NC) siRNA.


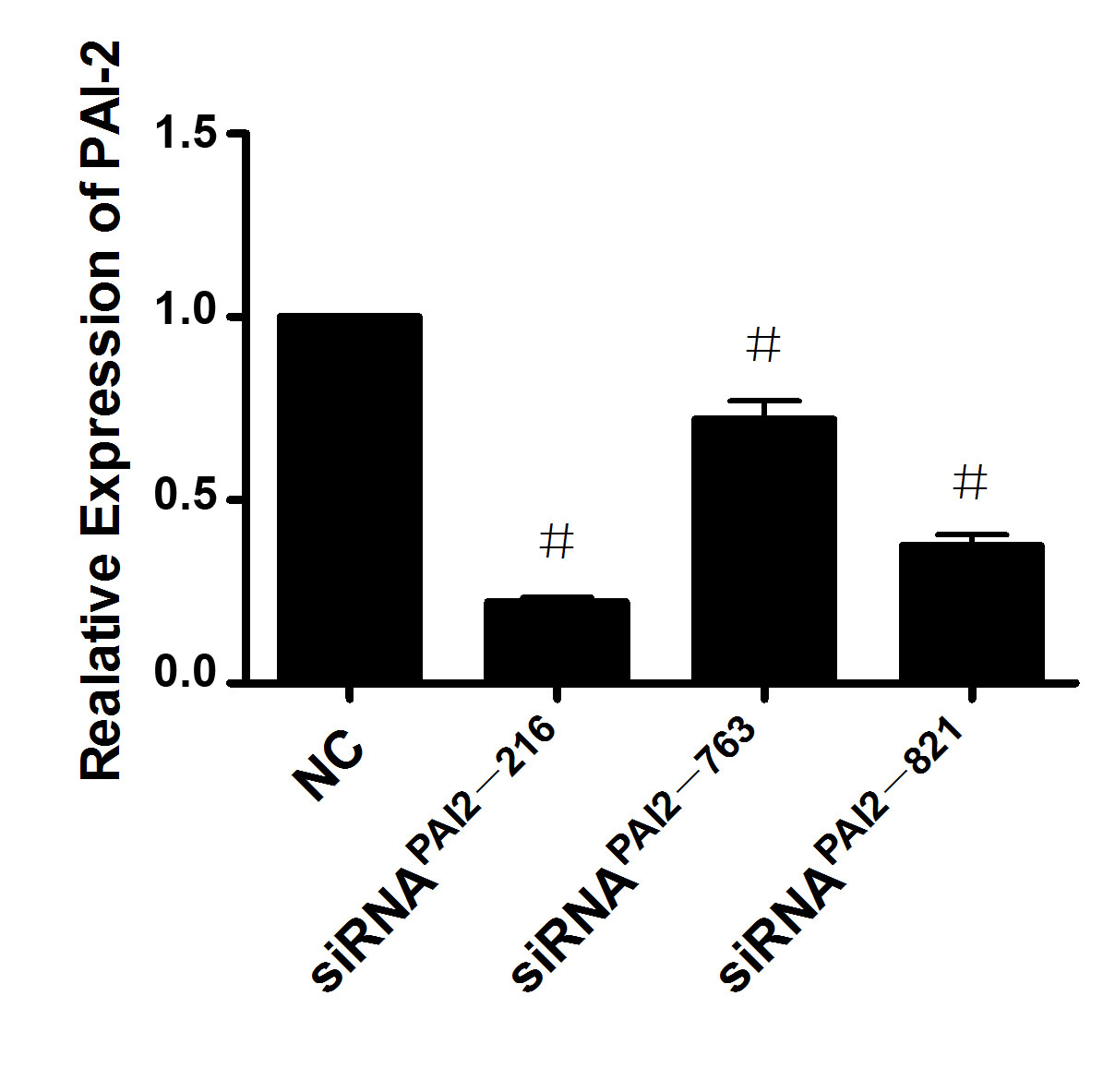


# Supplementary Figure 5

**Representative images of murine peritoneal macrophages were infected lentivirus carrying Akt and its mutants**.

# (A) Fluorescence microscopic images of 293FT cells transfected with lentivirus at 24 h after transfection ; (B) Fluorescence microscopic images of peritoneal macrophages at 72h after infected with Lentivirus.


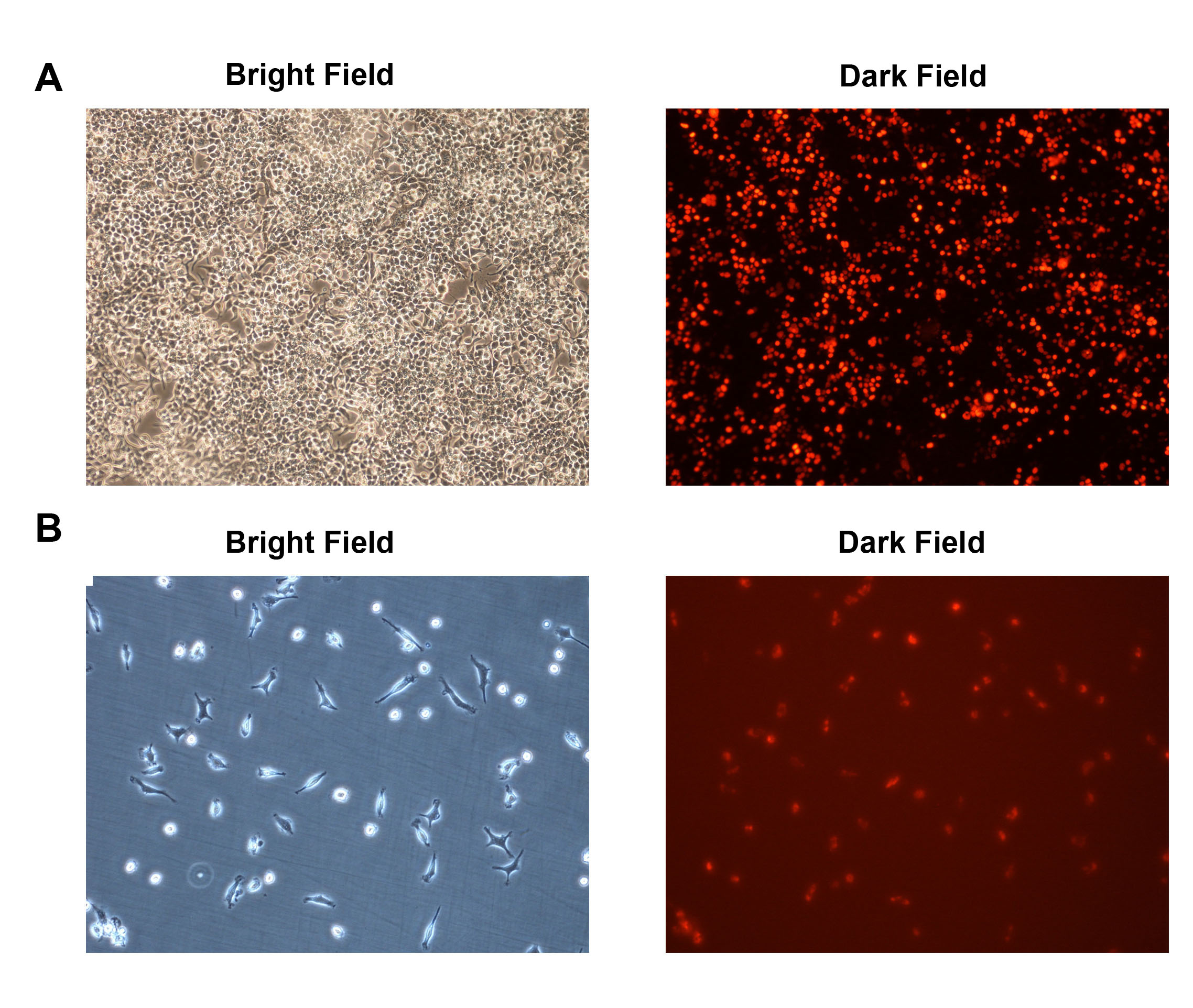


**Table S1 Primers sequences for plasmids construction**

| **Application** |  | **Primer Sequence** |
| --- | --- | --- |
| **AKT cloning** | forward | 5’- TAGGATCC AGCGACGTGGCTATTGTGAAG -3’ |
|  | reverse | 5’- TGAATTCTCAGGCCGTGCCGCTGG CCGAG -3’ |
| **AKT S473A mutan** | forward | 5’- GCCTACTCGGCCAGCGGCACG -3’ |
|  | reverse | 5’- GAACTGGGGAAGTGGGGCCTGC - 3’ |
| **AKT T308A mutan** | forward | 5’- GCCTTTTGCGGCACACCTGAGTACCT -3’ |
|  | reverse | 5’- CTTCATGG TGGCACCGTCCTTGATCC -3’ |
| **Subclone to lentiviral vector** | forward | 5’-ATGAGCGACGTGGCTATTGTGAAGGAGGGTT-3’ |
|  | reverse | 5’- GCTCTAGATCAGGCCGTGCCGCTGGCCGAG -3’ |
